# Fear From Afar, Not So Risky After All: Distancing Moderates the Relationship Between Fear and Risk Taking

**DOI:** 10.3389/fpsyg.2021.674059

**Published:** 2021-06-25

**Authors:** Lewend Mayiwar, Fredrik Björklund

**Affiliations:** ^1^Department of Leadership and Organizational Behaviour, BI Norwegian Business School, Oslo, Norway; ^2^Department of Psychology, Lund University, Lund, Sweden

**Keywords:** judgment and decision making, emotion regulation, psychological distance, cognitive reappraisal, incidental emotions, risk taking, self-distancing

## Abstract

A growing line of research has shown that individuals can regulate emotional biases in risky judgment and decision-making processes through cognitive reappraisal. In the present study, we focus on a specific tactic of reappraisal known as *distancing*. Drawing on appraisal theories of emotion and the emotion regulation literature, we examine how distancing moderates the relationship between fear and risk taking and anger and risk taking. In three pre-registered studies (*N*_*total*_ = 1,483), participants completed various risky judgment and decision-making tasks. Replicating previous results, Study 1 revealed a negative relationship between fear and risk taking and a positive relationship between anger and risk taking at low levels of distancing. Study 2 replicated the interaction between fear and distancing but found no interaction between anger and distancing. Interestingly, at high levels of distancing, we observed a reversal of the relationship between fear and risk taking in both Study 1 and 2. Study 3 manipulated emotion and distancing by asking participants to reflect on current fear-related and anger-related stressors from an immersed or distanced perspective. Study 3 found no main effect of emotion nor any evidence of a moderating role of distancing. However, exploratory analysis revealed a main effect of distancing on optimistic risk estimation, which was mediated by a reduction in self-reported fear. Overall, the findings suggest that distancing can help regulate the influence of incidental fear on risk taking and risk estimation. We discuss implications and suggestions for future research.

## Introduction

Studies in the last couple of decades have provided significant insight into the complex ways in which emotions influence judgments and decisions. Although emotions serve as sources of information that help individuals navigate through uncertainty, emotions can also “carry over” and influence judgments and decisions in a biasing way (Lerner et al., 2015). As a result, scientists have increasingly recognized the importance of identifying specific ways to minimize such biases (Lerner et al., 2015). While still in its infancy, an emerging and promising line of research has explored how various emotion regulation strategies influence risky decision making (Sokol-Hessner et al., 2009, 2013; Heilman et al., 2010; Miu and Crişan, 2011; Panno et al., 2013). The present study seeks to contribute to this developing line of research in several ways.

First and foremost, we examine a specific emotion regulation tactic that has received relatively little attention in judgment and decision-making research, namely, *distancing*. This tactic involves mentally changing the psychological distance of a stimulus to reduce its emotional impact (see Powers and LaBar, 2019). It has been associated with a range of emotional (Kross et al., 2014; Bruehlman-Senecal and Ayduk, 2015; Nook et al., 2017, 2020; Ahmed et al., 2018; Powers and LaBar, 2019; White et al., 2019) and cognitive benefits (Kross and Grossmann, 2012; Grossmann and Kross, 2014; Sun et al., 2018). Studies suggest that distancing requires less effort than other tactics and strategies, rendering it a promising tool in practical settings (Powers and LaBar, 2019). Second, the present study examines how distancing moderates the relationship between *incidental emotions*—emotions that are elicited from unrelated situations—and risk taking. Finally, we focus on specific emotions that can be expected to lead to opposite effects on risk; n, fear and anger (Lerner and Keltner, 2000, 2001; Lerner et al., 2015). It is worth emphasizing at the outset that in some situations, emotions can be highly adaptive. However, individuals might wish to down-regulate emotions where they can be expected to lead to judgments and decisions that are inconsistent with one's goals or values. Moreover, whether risk taking is beneficial or detrimental is not a question that we can answer in this study.

## Theory and Hypotheses

### Incidental Fear and Anger

As noted by Lerner et al. (2015), the majority of research on emotion and risky decision making has focused on valence (i.e., subjective feelings of pleasantness/unpleasantness). Valence-based models posit that emotions of the same valence (i.e., positive vs. negative emotions) have similar effects on risk perception. Appraisal theories, on the other hand, posit that emotions of the same valence can have opposite effects on judgments and decisions. Moving beyond dimensions of valence, the Appraisal Tendency Framework (ATF; Lerner and Keltner, 2000, 2001) focuses on distinct emotions (e.g., fear, anger, sadness, happiness) and their associated appraisals (i.e., evaluations of events and situations). Lerner and Keltner (2001) demonstrated that fear and anger, both of which are negative valence and high arousal (i.e., intense) emotions, have opposite effects on risky judgments and decisions due to their distinct underlying appraisals of certainty and control (Lerner and Keltner, 2001; Lerner et al., 2003; Habib et al., 2015; Ferrer et al., 2017; Wake et al., 2020). Fear reduces risk taking due to its appraisals of uncertainty and low personal control. In contrast, anger increases risk taking due to its appraisals of certainty and personal control (Lerner and Keltner, 2001).

Finally, studies that examine the influence of specific emotions like fear and anger on judgments and decisions usually adopt an *incidental emotion* approach. In contrast to integral emotions, which are elicited by the decision task at hand, incidental emotions are elicited by unrelated events that carry over to the decision-making process (for an in-depth distinction, see Västfjäll et al., 2016). For instance, anger triggered in one situation (e.g., anger stemming from bad traffic while driving to work) can carry over to influence judgments and decisions in unrelated settings (e.g., deciding to invest in a risky project without giving the decision sufficient thought). Unlike integral emotions which are “normatively defensible input to judgment and decision making” (Lerner et al., 2015, p. 803), incidental emotional influences are often unwanted.

### Psychological Distance and Emotion Regulation

Trope and Liberman (2010) define psychological distance as “the subjective experience that something is close or far away from self, here and now” (p. 440). Psychological distance has been found to decrease emotional intensity (van Boven et al., 2010), and appears to be particularly effective in regulating basic emotions such as fear and anger (Katzir and Eyal, 2013). In a study by Davis et al. (2011), participants who imagined that aversive images presented on a screen were moving further away from them exhibited lower negative affect and physiological responses. Adopting a temporally distant perspective from future stressors has been associated with lower levels of anxiety and image vividness (White et al., 2019). Supporting these findings, Nook et al. (2017) demonstrated that participants who wrote about negative images using psychologically distant (vs. close) language in physical, social, and temporal domains exhibited lower negative affect. Bruehlman-Senecal and Ayduk (2015) found that participants who reflected on how they would feel about recent stressors in the distant future showed significantly lower emotional distress. Moreover, the authors found that an impermanence focus (e.g., focusing on how one's feelings might change with time) mediated this effect. Similar results have been found in studies examining individual differences in temporal distancing (Bruehlman-Senecal et al., 2016). Not only do these findings support folk sayings like “time heals all wounds,” but they show that people can mentally project themselves into the future to reduce stressors in the here and now. Other studies have shown that distancing is also associated with cognitive benefits, such as wise reasoning (e.g., realizing the limits of one's knowledge and recognizing diverse perspectives; Kross and Grossmann, 2012; Grossmann and Kross, 2014). According to Construal Level Theory (CLT; Trope and Liberman, 2010), psychological distance exists across various dimensions, including temporal, social, and spatial distance. In terms of its emotion-regulatory function, it means that negative emotions can be downplayed by imagining that the emotional stimulus is temporally, physically, or socially far from the self. Indeed, distancing is a specific tactic of a general emotion regulation strategy known as *reappraisal* (see a taxonomy of distancing and emotion regulation by Powers and LaBar, 2019). Reappraisal involves changing one's mental representation of an emotion-eliciting stimulus to minimize its emotional impact. This can be done through either *reinterpretation* (e.g., thinking of a lay-off as an opportunity to pursue a more desirable career) or *distancing* (e.g., adopting the perspective of a distant, uninvolved participant when dealing with a personal conflict at work). Our review, however, is restricted to studies investigating the distancing tactic. Although both tactics have been found to be effective in regulating negative emotions, some evidence suggests that distancing is more effective than reinterpretation. For instance, Denny and Ochsner (2014) compared the effects of longitudinal training in distancing and reinterpretation. Compared to those who were trained in reinterpretation, participants who were trained in distancing showed lower levels of stress in daily life and were more likely to evaluate aversive content neutrally. Moreover, distancing seems to require less effort than reinterpretation because it does not target specific features of an emotion-eliciting stimulus (Moser et al., 2017). Thus, distancing may offer regulatory benefits across a broader range of situations. Although emotion regulation studies are typically restricted to the down-regulation of negative emotions, there are situations where one's goal might be to down-regulate positive emotions or up-regulate negative emotions (e.g., Tamir and Bigman, 2014; Tamir and Ford, 2009). For example, like anger, happiness can lead to excessive risk taking (Lerner and Keltner, 2001).

### Psychological Distance and Risk

Only recently have studies started to explore the role of psychological distance in risky decision making. This small set of studies has tested how psychological distance, across various dimensions, impacts risk taking (e.g., Polman, 2012; Raue et al., 2015; Sun et al., 2017; Zhang et al., 2017). For instance, social distance (i.e., choosing for socially distant others) has been associated with reduced loss aversion (Polman, 2012; Andersson et al., 2014; Sun et al., 2017; Zhang et al., 2017). In a medical scenario about a deadly virus, people who chose for others showed a greater tendency to accept the vaccine than those who chose for themselves (Zikmund-Fisher et al., 2006). Similar results have been obtained in studies examining temporal distance. Chandran and Menon (2004) showed that “every day” framing made risks appear more proximal and concrete than “every year” framing, resulting in increased risk perceptions, intentions to engage in preventive behavior, and increased anxiety about hazards. Raue et al. (2015) manipulated psychological distance by varying the temporal, social, and spatial distance in decision scenarios. Across several experiments with students, physicians, and hotel managers, psychological distance reduced framing effects. Finally, Sun et al. (2018) similarly demonstrated that self-distancing (by adopting a distant observer's perspective) reduced probability-weighting biases.

The influence of psychological distance on risk is believed to result from a reduction in emotional intensity, as distance enables individuals to “zoom out” and transcend features of the here and now (Fujita et al., 2016). This notion is consistent with studies that have linked self-distancing to enhanced wise reasoning (Kross and Grossmann, 2012; Grossmann and Kross, 2014). These findings raise an interesting question; how does psychological distance shape the role of emotions like fear in decisions and judgments involving risk? A recent line of research provides a starting point. Although, it appears that these studies have either examined the general strategy of reappraisal or reinterpretation, not distancing. A study by Heilman et al. (2010) examined incidental regulation of fear and disgust on risk taking in the Balloon Analog Risk Task (BART) and Iowa Gambling Task (IGT). Participants were instructed to either reappraise or suppress their emotions while watching a fear-inducing or disgust-inducing video. As predicted, Heilman et al. (2010) found that reappraisal effectively reduced the influence of these two incidental emotions in both tasks. Similar results have been reported in studies examining integral emotion regulation and risk taking. Sokol-Hessner et al. (2009) found that instructing participants to adopt the perspective of a trader promoted risk taking by reducing physiological arousal. Building on these findings (Panno et al., 2013) found the same pattern of results for habitual reappraisal (i.e., naturally occurring individual differences in reappraisal). Specifically, habitual reappraisal was related to increased risk taking, accompanied by decreased sensitivity to changes in probability and loss amount. Yet, no study has directly tested how the distancing tactic of reappraisal regulates the influence of incidental emotions on judgments and decisions involving risk. This might be of particular interest in light of the benefits of distancing discussed in the previous section.

## Present Research

Few studies have examined how psychological distance moderates the influence of incidental emotions on judgments and decisions involving risk. Some of the studies covered earlier have manipulated distance by varying the proximity to targets in risky decision-making tasks (Chandran and Menon, 2004; Raue et al., 2015; Sun et al., 2017; Zhang et al., 2017) or instructed participants to adopt a distant perspective while completing a task (Sun et al., 2018). The authors behind some of these studies speculate that the impact of psychological distance on risk occurs via a reduction in emotional intensity (e.g., Raue et al., 2015; Sun et al., 2018). The present study aims to test this hypothesis by examining how distancing moderates the relationship between incidental emotions and risky judgments and decisions. More specifically, we focus on the regulation of fear and anger. A comparison between fear and anger is of theoretical interest since both are characterized by negative valence and high arousal (Smith and Ellsworth, 1985), but differ in their underlying appraisals (i.e., mental evaluations of a situation). While fear is characterized by appraisals of uncertainty and lack of control, anger is characterized by the opposite appraisal patterns. The ATF predicts that, because of their different appraisal patterns, fear should decrease risk taking whereas anger should increase risk taking. Thus, we predict that the opposing effects of anger and fear on risk taking will be particularly strong at low levels of distancing. We believe that this approach can help provide a more nuanced understanding of the role of emotion regulation in decision making, by showing that the impact of emotion regulation on judgments and decisions might depend on the target emotion.

Taken together, our study set out to examine how distancing moderates the influence of fear and anger on risk taking. Following our pre-registered hypotheses, we hypothesized that distancing would moderate the negative relationship between fear and risk taking, and the positive relationship between anger and risk taking. We conducted three pre-registered and high-powered studies to test these hypotheses. Study 1 tested the moderating role of habitual distancing on the relationship between trait fear and anger on risk taking. Study 2 experimentally manipulated distancing to examine whether trait fear and trait anger exert stronger effects on risk taking when decision scenarios are imagined as proximal. In other words, Study 2 examined how distancing from the decision-making task regulates the influence of incidental (trait) emotions. Finally, Study 3 manipulated both emotions (fear and anger) and distancing to examine how distancing from current fear-related and anger-related stressors carries over to impact subsequent risk taking.

## Ethics and Transparency Statement

The three studies presented in this article received ethical approval from the Norwegian Center for Research Data (NSD) before data collection. Participants in each study provided their consent to participate. We report how we determined the sample size, all data exclusions, all manipulations, and all measures collected in this study (Simmons et al., 2012). We pre-registered each study on the Open Science Framework (OSF) prior to data collection. The pre-registrations, data, code, and materials associated with this paper are available on the OSF repository.^1^

## Study 1

### Method

#### Participants

A total of 400 participants were recruited from Amazon's Mechanical Turk (MTurk), using the CloudResearch platform that blocks low quality participants by default (Litman et al., 2017). MTurkers were eligible to participate only if they were currently residing in the US, were native English speakers, completed a minimum of 500 surveys, and had a 95% MTurk HIT approval rating. Participants were paid $1.20 for the roughly 10-min long study. Following the pre-registered exclusion criteria, the final sample included 370 participants (198 males, 171 females, 1 other/prefer not to answer; *M*_*age*_ = 41.58, *SD*_age_ = 11.96). Participants were excluded if they; spent <2 min on the entire survey, indicated low English proficiency, reported not being serious about filling in the survey, failed a bot check, failed two out of three attention checks, and if they had correctly guessed the purpose of the study. We estimated the sample size by performing an a-priori power analysis (using GPower 3.1.9.4) for a hierarchical linear regression model predicting risk preference. The power analysis indicated that we needed a sample of 355 participants to detect a small effect size (*f*^2^ = 0.05; based on a meta-analysis by Wake et al., 2020). We entered the effect size estimate into the power analysis with the following input parameters: α = 0.05, power = 0.90, number of tested predictors = 6.

#### Design and Procedure

Participants were randomly assigned to receive the risky decision-making tasks in either the gain frame or loss frame (see description below). At the start of the survey, they read a consent form and indicated their agreement. Those who agreed received a brief cover story to dissociate the emotion measures from the risk preference measures. Specifically, we told them that different researchers had pooled together their questions for efficiency purposes and that the survey contained two different questionnaires: a “Self-Evaluation” questionnaire and a second questionnaire about “Preferences.” The trait emotions and habitual distancing measures (and items) were presented first, in random order.

#### Measures

##### Habitual Distancing

Individuals' general tendency to engage in distancing to regulate negative emotions was measured using the single-factor Temporal Distancing Questionnaire, developed by Bruehlman-Senecal et al. (2016). Across eight statements, participants indicated how they typically respond to negative events by taking a broad and distant perspective (1 = “strongly disagree,” 7 = “strongly agree”). Example statements included “I generally don't take a step back from the event and place it in a broader perspective” (reverse-coded), “I focus on how my feelings about the event may change with time,” and “I think about how small the event is in the bigger picture of my life.” The scale demonstrated strong reliability (α = 0.88).

##### Trait Fear

Dispositional fear was measured using the Penn State Worry Questionnaire (PSWQ; Meyer et al., 1990). Responses were measured on a 7-point Likert scale (1 = “not at all typical of me,” 7 = “very typical of me”). All items were averaged to form a single variable. Example items included “If I do not have enough time to do everything, I do not worry about it” (reverse-coded), “My worries overwhelm me,” and “I have been a worrier all my life.” The PSWQ has been used in previous studies examining financial risk taking (Maner et al., 2007). The scale demonstrated strong reliability (α = 0.97). Although some theorists conceptualize worry and fear as two different (albeit very similar) emotions (Öhman, 2008), the present study follows the common, broader conceptualization of fear as an emotion that encompasses worry and anxiety (e.g., Borkovec et al., 1998). Indeed, studies on fear and risk taking typically operationalize fear using measures of anxiety and worry. Furthermore, a recent meta-analysis by Wake et al. (2020) found no differences in the relationship between emotion and risk taking between studies that referred to “fear” and those that referred to “anxiety.”

##### Trait Anger

We measured trait anger using the State-Trait Anger Expression Inventory (STAXI-II; Spielberger, 1999). Using a 10-item scale, participants rated the extent to which various behaviors were typical of them (1 = “almost never,” 4 = “almost “always”). Items were averaged to form a single trait anger variable. The STAXI-II is commonly used in studies examining emotions and risk taking (Lerner and Keltner, 2001; Gambetti and Giusberti, 2012, 2014). The scale demonstrated strong reliability (α = 0.90).

##### Risky Decision-Making Tasks

Participants were presented with three different framing problems that were modeled on the classic Unusual Disease Problem (Kahneman and Tversky, 1979)^2^: The Cancer Problem (Fagley and Miller, 1987), Plant Problem (Bazerman, 1984), and the Shareholding Problem (Teigen and Nikolaisen, 2009). Half of the participants received the three risky decision-making tasks in the gain frame, while the other half received them in the loss frame. In each task, participants read a scenario and indicated the extent to which they preferred one option over the other on a 7-point Likert scale (1 = “strongly prefer option A over option B,” 7 = “strongly prefer option B over A”). Option A was always the safe option, and option B the risky option. Thus, for each participant, risk preference was measured three times. A full description of these tasks can be found on the OSF repository (see text footnote 1). For example, in the Plant Problem (adapted from Bazerman, 1984), participants read:

A large hi-tech company is experiencing serious economic troubles and needs to lay off 6,000 employees. The vice president has been exploring alternative ways to avoid this crisis and has developed two plans:
(*gain frame*)
Plan A: This plan will save 2,000 jobs.
Plan B: This plan has a 1/3 probability of saving all 6,000 jobs, but a 2/3 probability of saving no jobs.
(*loss frame*)
Plan A: This plan will result in the loss of 4,000 jobs.
Plan B: This plan has a 2/3 probability of resulting in the loss of all 6,000 jobs, but a 1/3 probability of losing no jobs.

###### Control Variables.

Following the pre-registration, age and gender were included as control variables. Previous research has found that males are more likely to engage in risky behavior and to respond to anger with risk taking (Ferrer et al., 2017). Furthermore, risk taking has also been found to decrease with age (Rolison et al., 2014). We also controlled for framing condition (0 = Gain frame, 1 = Loss frame) to account for potential differences in the influence of emotions in gain and loss frames. The subsequent studies use the same control variables.^3^

#### Statistical Analysis

A linear hierarchical multilevel model was fitted using the lme4 (Bates et al., 2014) and the lmerTest packages implemented in RStudio (R Core Team, 2014). Risk preference was predicted by the experimental manipulation (gain vs. loss frame), dispositional fear and anger, habitual distancing, and the interaction of habitual distancing with dispositional fear and anger. Participants and decision tasks were treated as random-intercept effects. The discussion will only focus on the final, overall model (i.e., Step 3). However, mean-centered beta coefficients and model fit statistics for each step of the regression are listed in Table 1. The choice of a linear mixed model deviated from the pre-registration, which specified the use of hierarchical multiple regression. A linear mixed model seemed more appropriate, however, as it accounts for repeated-measures dependencies—in this case, the repeated measure of risk preference across the three risky decision-making tasks. The results remain the same regardless of the analytical approach used. Assumptions of normality of residuals, linearity, and heteroscedasticity did not seem to be violated. For this and the two subsequent experiments, one-tailed *p*-values and confidence intervals are reported for the pre-registered directional hypotheses (Cho and Abe, 2013).^4^ For all other tests, two-tailed *p*-values are reported. Descriptive statistics of key variables across the three studies can be found in the online repository (see text footnote 1).

**Table 1:** Summary of hierarchical linear mixed model analysis predicting risk taking (Study 1).

	**Model 1**		**Model 2**		**Model 3**	
**Predictors**	**Estimates**	**CI**	**Estimates**	**CI**	**Estimates**	**CI**
Intercept	3.17^**^	2.73–3.61	3.18^**^	2.75–3.62	3.18^**^	2.74–3.62
Age	−0.01	−0.02–0.00	−0.02	−0.02–0.01	−0.01	−0.02–0.00
Gender	−0.14	−0.42–0.14	−0.17	−0.45–0.12	−0.16	−0.45–0.12
Framing	0.43^**^	0.16–0.71	0.43^**^	0.16–0.71	0.44^**^	0.17–0.72
Anger			0.17	−0.08–0.42	0.06	−0.21–0.32
Fear			0.04	−0.10–0.17	0.05	−0.08–0.18
Distancing			0.13	−0.00–0.26	0.10	−0.03–0.24
Distancing × Anger					−0.25^*^	−0.46 to −0.03
Distancing × Fear					0.10^*^	0.01–0.20
**Random Effects**						
σ^2^	2.12		2.12		2.12	
τ_00_	1.13_subject_		1.11_subject_		1.08_subject_	
	0.11_scenario_		0.11_scenario_		0.11_scenario_	
ICC	0.37		0.36		0.36	
N	369_subject_		369_subject_		369_subject_	
	3_scenario_		3_scenario_		3_scenario_	
Observations	1,107		1,107		1,107	
Marginal *R^2^*/Conditional *R^2^*	0.018/0.379		0.024/0.379		0.031/0.379	

*Continuous predictors are mean-centered*.

* *p < 0.05*,

** *p < 0.01*.

*One-tailed p-values and CIs are reported for the two hypothesized relationships (fear, anger, and their interactions with distancing)*.

*σ^2^, within-person variance; τ_00_, between-person variance; CI, confidence interval; ICC, intraclass correlation*.

### Results

#### Hypotheses Testing

All continuous predictors were mean centered before running the analyses (Aiken et al., 1991). Adding “subject” and “scenario” as random effects significantly improved the model fit compared to the model without the random effects, supporting the rationale for using a mixed model. The results from the hierarchical multilevel analysis are summarized in Table 1.^5^ Risk preference was significantly higher in the loss frame, β = 0.44, *p* = 0.001 (two-tailed), 95% CI [0.17, 0.72], thus, replicating the classic framing effect. Supporting the pre-registered directional moderation hypotheses, the final model indicated that habitual distancing significantly interacted with dispositional fear, β = 0.10, *p* = 0.038 (one-tailed), 90% CI [0.01, 0.20] and anger, β = −0.25, *p* = 0.029 (one-tailed), 90% CI [−0.46, −0.03] in the predicted directions. None of the simple slopes for the interaction between fear and distancing (low distancing: β = −0.07, *p* = 0.51, high distancing: β = 0.16, *p* = 0.11) and the interaction between anger and distancing (low distancing: β = 0.34, *p* = 0.05, high distancing: β = −0.23, *p* = 0.38) were significant. Moreover, contrary to our predicted main effects of fear and anger, neither dispositional fear nor anger alone predicted risk preference (fear: β = 0.05, *p* = 0.28 (one-tailed), 90% CI = −0.08, 0.18; anger: β = 0.06, *p* = 0.36 (one-tailed), 90% CI = −0.21, 0.32).

**Figure 1:**
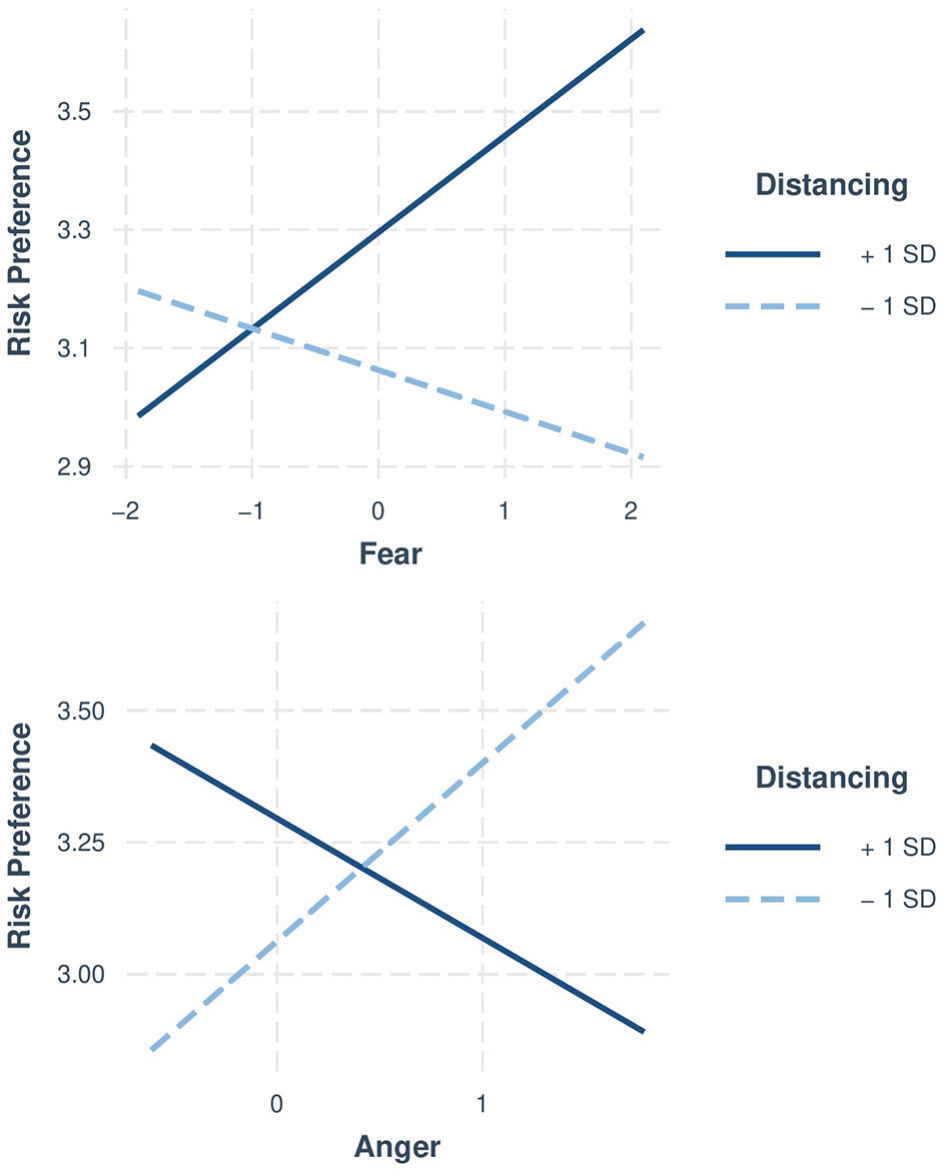
Significant moderation by distancing in Study 1. Upper panel: negative relationship between fear and risk taking at lower levels of distancing. Lower panel: positive relationship between anger and risk taking at lowers levels of distancing. Each interaction plot presents the relationship at two levels of the moderator variable (−1SD standard deviation and +1SD standard deviation). Risk preference scored on a 1–7 scale.

As shown in Figure 1,^6^ for individuals low on habitual distancing, dispositional fear is negatively related to risk preference whereas dispositional anger is positively related to risk preference (see text footnote 5). Interestingly, this pattern is reversed for individuals high on habitual distancing. Specifically, at high levels of distancing, fear is *positively* related to risk preference whereas anger is *negatively* related to risk preference. Thus, not only did distancing attenuate the relationship between fear and risk preference, but even reversed the relationship. These results are discussed later in the Discussion section.

Finally, following the pre-registered exploratory analyses, we also tested whether the interactions depended on the framing condition. Accordingly, a new model was tested that included two three-way interactions (fear^*^distancing^*^frame, anger^*^distancing^*^frame). None of the three-way interactions were significant (fear^*^distancing^*^frame: β = −0.11, *p* = 0.383 (two-tailed), 95% CI = −0.34, 0.13; anger^*^distancing^*^frame: β = 0.23, *p* = 0.398 (two-tailed), 95% CI = −0.30, 0.76). This is consistent with Lerner and Keltner (2001), who argued that the opposite effects of fear on anger (i.e., fear increasing risk aversion and anger increasing risk taking) should hold regardless of framing.

### Discussion

Study 1 examined whether habitual distancing (i.e., individuals' general tendency to adopt an objective and distant perspective when faced with negative events) moderates the influence of dispositional fear and anger on risk taking. Drawing on the ATF (Lerner and Keltner, 2001) and a developing line of research on emotion regulation and decision making (e.g., Heilman et al., 2010; Miu and Crişan, 2011; Panno et al., 2013), it was predicted that fear would be negatively related—and anger positively related—to risk taking, but only for individuals low on habitual distancing. Results supported both hypotheses. For individuals low on habitual distancing, fear decreased risk taking and anger increased risk taking. Interestingly, as opposed to the expected pattern of results, we found that fear *increased* risk taking whereas anger *decreased* risk taking at high levels of distancing. Although these results are difficult to interpret, one might speculate that people who naturally engage in distancing are more likely to reframe decision problems in a way that alters the influence of incidental emotions. We suggest that future studies aim to uncover underlying mechanisms. Consistent with Lerner and Keltner (2001), these results did not depend on the frame that participants received. Moreover, dispositional fear and anger alone did not predict risk taking. Their associations with risk taking were qualified by distancing. Finally, it is also worth mentioning that this study included three different domains of risk, thus accounting for possible domain-specific variations (Kühberger et al., 1999). Taken together, the results suggest that dispositional emotions and emotion regulation through distancing can predict the decisions people make. In Study 2, we used new measures of fear and anger to examine whether the null findings might be attributed to the measures.

## Study 2

Study 2 attempted to address some of the limitations in Study 1 in two ways. First, we included new measures of dispositional fear and anger. Second, instead of measuring habitual distancing, we manipulated distancing. Because dispositional emotions may be particularly difficult to regulate (Lerner and Keltner, 2001), an interesting question is whether manipulating distancing from the risky decision-making task itself can reduce the influence of such emotions. To this end, Study 2 aimed to test whether distancing moderates the relationship between (1) dispositional fear and risk taking and (2) dispositional anger and risk taking.

### Method

#### Participants

A total of 600 participants were recruited from MTurk, using the CloudResearch platform (Litman et al., 2017). The sample size was estimated by performing an a-priori power analysis (using GPower 3.1.9.4) for a hierarchical linear regression model predicting risk preference. The power analysis indicated that we needed a sample of 550 participants to detect a small effect size (*f*^2^ = 0.02; based on a meta-analysis by Wake et al., 2020). The effect size estimate was entered into the power analysis with the following input parameters: α = 0.05, power = 0.80, number of tested predictors = 3. MTurkers were eligible to participate only if they were currently residing in the US, were native English speakers, completed a minimum of 500 surveys, and had a 95% MTurk HIT approval rating. Participants were paid $1.30 for the roughly 10-min long study. As specified in the pre-registration, participants were excluded if they; spent <2 min on the entire survey, indicated low English proficiency, reported not being serious about filling in the survey, failed a bot check, and if they correctly guessed the purpose of the study. Although not specified in the pre-registration, participants were also excluded if they spent <3 s on the page that included the self-distancing instructions. The final sample included 470 participants (235 males, 233 females, 2 other/prefer not to answer; *M*_*age*_ = 40.55, *SD*_*age*_ = 12.21). This study received ethical approval from the Norwegian Center for Research Data (NSD) before data collection.

#### Design and Procedure

This study used a 2 (distance: near vs. far) x 2 (frame: gain vs. loss) between-subjects design. As in Study 1, participants read a consent form and indicated their agreement. Those who agreed went on to receive a similar cover story and answered the trait emotions measurements. Again, these measures (and items) appeared in random order.

#### Measures

##### Self-Distancing Manipulation

Participants were randomly assigned to receive either a low distance or high distance prompt right before the risky decision-making tasks were presented. In the high distance condition, participants were instructed to “Imagine that the situation in the scenario happened very far from where you are now, like very long ago, very far in the future, or in another distant country.” In the low distance condition, participants were instructed to “Imagine that the situation in the scenario happened very close to where you are now, like yesterday, tomorrow, or right in front of your eyes.” This manipulation was adapted from van Dijke et al. (2018) (for a similar distancing manipulation, see Sun et al., 2018).

##### Trait Fear

Trait fear was measured using the Fear Survey Schedule-II (Geer, 1965; Bernstein and Allen, 1969). Responses were measured on a 7-point Likert scale (1 = “no fear,” 7= “terror”). All items were averaged to form a single variable. Example items included “I fear being criticized,” “I'm afraid of snakes,” and “I'm afraid of not being a success.” This scale has been widely used in previous studies examining fear and risk taking (e.g., Lerner and Keltner, 2001). The scale demonstrated strong reliability (α = 0.86).

##### Trait Anger

We used two complementary measures of trait anger: the State-Trait Anger Expression Inventory (STAXI-II; Spielberger, 1999) and Lerner and Keltner's (2001) 10-item anger scale. We combined the two measures to form one single index of trait anger (α = 0.94) Subjects rated the extent to which various behaviors were typical of them. Example items from the STAXI-II included “I am quick tempered” and “I feel infuriated when I do a good job and get a poor evaluation.” Example items from the Lerner and Keltner (2001) anger scale included “I often find myself feeling angry” and “Other drivers on the road infuriate me.” Responses were measured on a 7-point Likert scale (1 = “not at all true of me,” 7 = “very true of me”).

##### Risky Decision-Making Tasks

We used the same risky decision-making tasks as those in Study 1. Participants were randomly assigned to receive the tasks in either the gain frame or loss frame.

##### Manipulation Check

We used a single item from van Dijke et al. (2018): “How far away from the described scenarios did you feel?” (1 = “very close” to 9 = “very far”). Participants received the manipulation check after the decision-making task.

#### Statistical Analysis

Following our pre-registered plan, before proceeding to our main analysis of the interaction between distancing and emotions, we ran a two-way ANOVA to examine whether there was an interaction between framing and distancing in predicting risk preference. Specifically, we predicted that risk preference would be higher in loss frames and lower in the gain frame when distance is low. The ANOVA yielded a main effect of framing, *F*_(1, 466)_ = 52.51, *p* < 0.001, $\eta_{\text{p}}^{2}$ = 0.101. However, the ANOVA yielded no main effect of distancing, *F*_(1, 466)_ = 0.71, *p* = 0.401, $\eta_{\text{p}}^{2}$ = 0.001, and no interaction between distancing and framing, *F*_(1, 466)_ = 0.88, *p* = 0.35, $\eta_{\text{p}}^{2}$ = 0.002.

Next, we proceed with our main analysis to examine the interaction between fear and distancing, and anger and distancing. A linear hierarchical multilevel model was fitted using the lme4 (Bates et al., 2014) and the lmerTest packages implemented in the R statistical environment (R Core Team, 2014). As in Study 1, the decision to use multilevel analysis deviated from the pre-registration, but results remain the same regardless of the analytical approach. Risk preference was predicted by framing (0 = Gain 1 = Loss), dispositional fear and anger, distancing (−0.5 = Near, +0.5 = Far), and the interactions of distancing with dispositional fear and anger. We used effect-coding (−0.5/+0.5) instead of dummy coding (1/0) to be able to interpret the lower-order main effects (Singmann and Kellen, 2019). Participants and decision scenario were treated as random-intercept effects. The discussion will focus only on the final, overall model (i.e., Step 3). Mean-centered beta coefficients and model fit statistics for each step of the regression are listed in Table 2. Assumptions of normality, linearity, and heteroscedasticity did not appear to be violated.

**Table 2:** Summary of hierarchical linear mixed model analysis predicting risk taking (Study 2).

	**Model 1**		**Model 2**		**Model 3**	
**Predictors**	**Estimates**	**CI**	**Estimates**	**CI**	**Estimates**	**CI**
Intercept	3.49^**^	3.23–3.76	3.48^**^	3.20–3.76	3.47^**^	3.20–3.75
Age	0.01	−0.00–0.01	0.01	−0.00–0.02	0.01	−0.00–0.02
Gender	−0.23^*^	−0.43 to −0.03	−0.24^*^	−0.45 to −0.04	−0.25^*^	−0.46 to −0.05
Framing	0.71^**^	0.52–0.91	0.69^**^	0.50–0.88	0.71^**^	0.52–0.90
Distance			0.07	−0.12–0.28	0.07	−0.12–0.26
Anger			0.18***	0.09–0.27	0.20^**^	0.08–0.32
Fear			0.01	−0.07–0.10	−0.12	−0.24–0.01
Distance × Anger					−0.04	−0.21–0.13
Distance × Fear					0.25^*^	0.08–0.42
**Random Effects**						
σ^2^	2.04		2.04		2.04	
τ_00_	0.47_subject_		0.43_subject_		0.41_subject_	
	0.05_scenario_		0.05_scenario_		0.05_scenario_	
ICC	0.20		0.19		0.19	
N	468_subject_		468_subject_		468_subject_	
	3_scenario_		3_scenario_		3_scenario_	
Observations	1,404		1,404		1,404	
Marginal *R^2^*/Conditional R^2^	0.053/0.247		0.069/0.247		0.075/0.247	

*Continuous predictors are mean-centered*.

* *p < 0.05*,

** *p < 0.01*.

*One-tailed p-values and CIs are reported for the hypothesized relationships (fear, anger, and their interactions with distancing)*.

*σ^2^, within-person variance; τ_00_, between-person variance; CI, confidence interval; ICC, intraclass correlation*.

### Results

#### Manipulation Check

An independent samples *t*-test revealed that participants in the far condition imagined the decision scenarios to be further away (*M* = 8.13, *SD* = 1.13) than participants in the close condition (*M* = 2.24, *SD* = 1.60), *t*(468) = −46.14, *p* < 0.001, *d* = −4.27, 95% CI [−4.58, −3.93].

#### Hypotheses Testing

All continuous predictors were mean-centered before running the analyses (Aiken et al., 1991). Including “subject” and “scenario” random effects significantly improved the model fit compared to the model without the random effects, supporting the rationale for using a mixed model. The results from the hierarchical multilevel analysis are summarized in Table 2. Risk preference was significantly higher in the loss frame, β = 0.71, *p* < 0.001, 95% CI [0.52, 0.90]. Thus, replicating the classic framing effects. Dispositional anger predicted higher risk taking, β = 0.20, *p* = 0.003 (one-tailed), 90% CI [0.07, 0.31]. Dispositional fear, on the other hand, did not significantly predict risk taking, although it was in the predicted direction, β = −0.12, *p* = 0.06 (one-tailed), 90% CI [−0.24, 0.01]. As predicted, distancing significantly interacted with fear, β = 0.25, *p* = 0.007 (one-tailed), 90% CI [0.08,0.42]. However, there was no interaction with dispositional anger, β = −0.04, *p* = 0.34 (one-tailed), 90% CI [−0.21, 0.13]. The simple slopes for the interaction between fear and distancing were not significant (low distance: β = −0.12, *p* = 0.12; high distancing: β = 0.13, *p* = 0.07).

**Figure 2:**
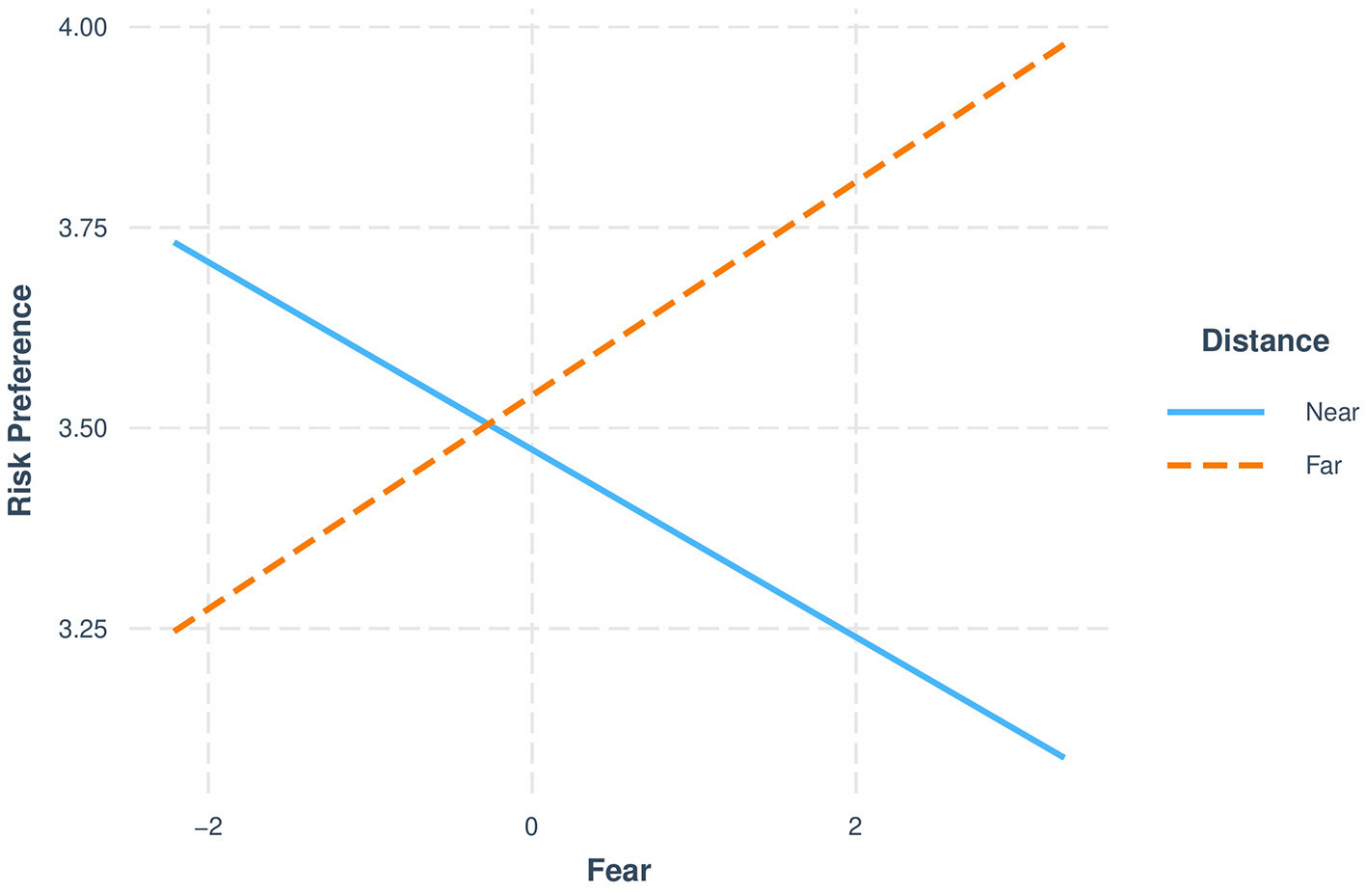
Significant moderation by distancing in Study 2. The interaction plot presents the relationship at two levels of the moderator variable (−1SD standard deviation and +1SD standard deviation). Risk preference scored on a 1–7 scale.

Figure 2 illustrates a cross-over interaction between dispositional fear and distancing. In the immersed condition, dispositional fear is negatively related to risk preference. In the distanced condition, dispositional fear is positively related to risk preference.

As in Study 1, pre-registered exploratory analyses were performed to test whether the two interactions depended on the framing condition. A new model was tested that included two three-way interactions (fear^*^distancing^*^frame and anger^*^distancing^*^frame). None of the three-way interactions were significant (fear^*^distancing^*^frame: β = 0.01, *p* = 0.95, 95% CI = −0.38, 0.41; anger^*^distancing^*^frame: β = −0.09, *p* = 0.66, 95% CI = −0.49, 0.31). However, we did not calculate power for these exploratory interactions, which needs to be taken into account when interpreting the results.

### Discussion

Study 2 extended Study 1 in two ways; (1) by including new measures of dispositional fear and anger, and (2) by manipulating distancing. As in Study 1, fear alone did not predict risk taking. However, anger was significantly and positively related to risk taking. This suggests that the main association between trait emotions and risk taking may depend on the specific measures used. The main hypothesis of interest was, however, the moderating role of distancing. In Study 2, we tested whether instructing individuals to distance themselves from the risky decision scenarios moderates the relationship between (1) dispositional fear and risk taking and (2) dispositional anger and risk taking. Consistent with Study 1, fear was negatively related to risk taking in the immersed condition. Interestingly, again, distancing not only attenuated this relationship but even reversed it, such that fear was *positively* related to risk-seeking in the distanced condition. Anger, on the other hand, did not interact with distancing. Finally, as in Study 1, neither interaction depended on the framing (i.e., loss vs. gain).

## Study 3

Study 3 attempted to replicate the previous findings in an experiment by manipulating both emotions and distancing. The aim was to test whether distancing oneself moderates the influence of fear and anger on risky judgments and decisions. Specifically, participants adopted either an immersed or distanced perspective while reflecting on fear-related and anger-related stressors before the risky judgment and decision-making tasks. Participants were not instructed to engage in distancing during the tasks as in Study 2. Rather, what we study here can be referred to as *incidental* distancing.

### Method

#### Participants

A total of 700 participants were recruited from MTurk, using the CloudResearch platform (Litman et al., 2017). We estimated the sample size by performing an a-priori power analysis (using GPower 3.1.9.4) for a two-way between subject ANCOVA. The power analysis indicated that we needed a sample of 603 participants to detect a small effect size of *f* ^2^ = 0.135 (based on a meta-analysis by Wake et al., 2020). The effect size estimate was entered into the power analysis with the following input parameters: α = 0.05, power = 0.80, number of groups = 4, number of covariates = 2. MTurkers were eligible to participate only if they were currently residing in the US, were native English speakers, completed a minimum of 500 surveys, and had a 98% MTurk HIT approval rating. Participants were paid $1.20 for the roughly 10-min long study. As specified in the pre-registration, participants were excluded if they; spent <2 min on the entire survey, indicated low English proficiency, reported not being serious about filling in the survey, failed a bot check and an attention check, and if they had correctly guessed the purpose of the study. The final sample included 643 participants (309 males, 328 females, 6 other/prefer not to answer; *M*_age_ = 41.27, *SD*_age_ = 13.15).

#### Procedure and Design

Study 3 used a 2 (emotion: fear vs. anger) × 2 (perspective: immersed vs. distanced) between-subjects design. Participants read a consent form first, and those who agreed proceeded to receive a similar cover story like the ones used in the previous two studies.

##### Emotion Induction

The emotion induction procedure was adapted from Lerner and Keltner (2001) and Lerner et al. (2003). The procedure consisted of two parts. First, they read a short story (131 words in the fear condition, 148 words in the anger condition) that described how the COVID-19 pandemic has increased unemployment and job loss (fear condition) or how the pandemic has resulted in unfair treatment of employees (anger condition). Below the paragraph were real news headlines that matched the content of the story. For instance, in the fear condition, participants saw news headlines about increased unemployment rates and job loss due to the pandemic. In the anger condition, participants saw headlines about companies that had taken advantage of the pandemic and treated employees in unethical ways. Materials are available on the OSF project page (see text footnote 1). In the second part, we asked the participants to think about a specific aspect of the pandemic that has made them most angry/afraid.

##### Self-Distancing Manipulation

Right after the emotion induction page, participants were asked to reflect on their thoughts and feelings about the emotional event that they identified on the previous page from an immersed or a distanced perspective (adapted from Bruehlman-Senecal and Ayduk, 2015, White et al., 2019). This manipulation focuses on the temporal dimension of psychological distance. Participants received the following instructions:

Immersed condition:
“Now that you've thought of a specific event related to the pandemic that makes you afraid [angry], imagine this very event unfold through your own eyes as if it was happening to you right now. As you continue to see the situation unfold in your own eyes, please take the next couple of minutes to describe your stream of thoughts about how you feel about this event that makes you afraid [angry].”
Distanced condition:
“Now that you've thought of a specific event related to the pandemic that makes you afraid [angry], take a few steps back and move away from the event to a point where it feels very distant from you. To help you do this, imagine what your life will be like 10 years in the future, envisioning what you might be doing and how you might be spending your time at this future time point.”

We told them to take at least 3 min to describe their current thoughts and feelings (participants could not proceed to the next page until 3 min had passed).

#### Measures

##### Risky Judgment and Decision-Making Tasks

This study included two risk operationalizations; risk taking and risk estimation. We measured risk preference using the same scale as in the previous two studies. This time, as per the pre-registration, participants were given only one risky decision-making task; the Plant Problem (Bazerman, 1984), in the gain frame. Our decision to use only the gain frame was based on a recent meta-analysis by Wake et al. (2020) that suggested a stronger relationship between fear and risk in gain frames.

Risk estimation was measured with an adapted version of Lerner's shortened optimistic risk estimation scale (Lerner and Keltner, 2001; Winterich et al., 2010). Participants indicated from 1 (extremely unlikely) to 7 (extremely likely) the likelihood that each of five positive and negative events would happen to them at any point in their future life. We slightly modified the scale in this study to ensure that the items were better suited for an MTurk sample. Specifically, we excluded the items “I had a heart attack before age 50” and “I got into a prestigious internship program.” These two items were replaced with an item from the original scale. The items included in this study were: 1. “I could not find a job for 6 months” (reverse-scored). 2. “I received statewide recognition in my profession.” 3. “My income doubled within 10 years after my first job.” 4. “I chose the wrong profession” (reverse-scored). 5. “I married someone wealthy.” Items were averaged to form an optimistic risk estimates score (α = 0.56). This indicates low reliability but is in line with previous studies (Winterich et al., 2010; Drace and Ric, 2012). As specified in our pre-registration, we included risk estimation as an additional measure to match our experiment more closely with Lerner and Keltner (2001, Study 4). Specifically, in their initial study examining trait fear and anger, they used the Unusual Disease Problem (see text footnote 2). However, in their follow-up experiment that manipulated both emotions, they used the risk estimation scale. We suspected that the influence of manipulated incidental emotions on risk taking might be weaker in decision tasks like the Plant Problem that seem somewhat more cognitively demanding. Unlike such decision tasks, the risk estimation scale concerns individuals' perceived likelihood of future events. This makes it possible for people to “guess” and rely on their intuition when estimating the likelihood of events—they simply do not have much else to base their judgments on than their gut feeling.

##### Manipulation Checks

To measure the effectiveness of emotion induction, participants were instructed to indicate how they felt while reflecting on the event in the writing task that they completed before the risky judgment and decision-making tasks. Participants rated the extent to which they felt fearful, worried, anxious, angry, outraged, and irritated (1= “not at all,” 7 = “very much”). The first three items were averaged to form an index for fear, and the last three items were averaged to form an index for anger. The temporal distancing manipulation check was measured with a single item: “To what extent did your thoughts during the reflection period focus on the present/near future vs. distant future?” (1 = “the present/near future,” 9 = “distant future”). This manipulation check was adapted from Bruehlman-Senecal and Ayduk (2015). Participants received the emotion and distance manipulation check items at the end of the survey.

### Results

#### Manipulation Checks

To examine whether our manipulations were successful, we ran a series of ANOVAs. For perceived distance, an ANOVA revealed that participants in the distant condition focused on the distant future (*M* = 6.07, *SD* = 1.36) more than participants in the immersed condition (*M* = 2.02, *SD* = 1.23), *F*_(1, 641)_ = 1,563.23, *p* < 0.001, $\eta_{\text{p}}^{2}$ = 0.710. For self-reported fear, a two-way ANOVA revealed a significant interaction between emotion and distancing conditions, *F*_(1, 639)_ = 23.94, *p* < 0.001, $\eta_{\text{p}}^{2}$ = 0.040. Tukey-adjusted pairwise *t*-tests indicated that participants in the immersed fear condition experienced more fear (*M* = 5.30, *SD* = 1.48) than participants in the distant fear condition (*M* = 3.21, *SD* = 1.99), *t*(639) = 10.64, *p* < 0.0001 (two-tailed), *d* = 1.18, 95% CI [0.94, 1.41], and the immersed anger condition (*M* = 3.91, *SD* = 1.90), *t*(639) = 7.02, *d* = 0.78, *p* < 0.0001 (two-tailed), 95% CI [0.55, 1.00]. For self-reported anger, a two-way ANOVA did not reveal a significant interaction between emotion and distancing conditions, *F*_(1, 639)_ = 0.53, *p* = 0.470, $\eta_{\text{p}}^{2}$ < 0.001. Suggesting that the manipulation worked in the intended way, Tukey-adjusted pairwise *t*-tests indicated that participants in the immersed anger condition experienced more anger (*M* = 5.58, *SD* = 1.41) than participants in the distant anger (*M* = 4.22, *SD* = 1.99), *t*(639) = 7.20, *p* < 0.0001 (two-tailed), *d* = 0.82, 95% CI [0.58, 1.05] and the immersed fear conditions (*M* = 3.16, *SD* = 1.73), *t*(639) = −13.08, *p* < 0.001 (two-tailed), *d* = −1.45, 95% CI [−1.69, −1.20]. Overall, these results suggest that the emotion and distancing manipulations were successful.

#### Hypotheses Testing

Two two-way ANCOVAs were performed that examined the effects of distancing and emotion on risk preference and optimism while controlling for age and gender. First, a two-way ANCOVA was tested with risk preference (from the framing problem) as the dependent variable. The main effects of emotion, *F*_(1, 636)_ = 0.00, *p* = 0.96, $\eta_{G}^{2}$ < 0.001, and distancing, *F*_(1, 636)_ = 2.06, *p* = 0.15, $\eta_{G}^{2}$ = 0.003, and their interactions were not significant, *F*_(1, 636)_ = 0.94, *p* = 0.33, $\eta_{G}^{2}$ = 0.001. A second two-way ANCOVA was performed with risk estimation as the dependent variable. The main effect of emotion, *F*_(1, 636)_ = 0.10, *p* = 0.76, $\eta_{G}^{2}$ < 0.001, and the interaction between emotion and distance, *F*_(1, 636)_ = 0.27, *p* = 0.60, $\eta_{G}^{2}$ < 0.001, were not significant. Incidental distancing, however, had a main effect on risk estimation, *F*_(1, 636)_ = 7.81, *p* = 0.005, $\eta_{G}^{2}$ = 0.01. Participants in the immersed condition (*M* = 3.16, *SD* = 1.10) were less optimistic in their risk estimates than participants in the distant condition (*M* = 3.42, *SD* = 1.15), *t*(638) = −2.82, *p* = 0.005 (two-tailed), *d* = −0.22, 95% CI [−0.38, −0.07]. As per the pre-registration, we also tested the difference in risk estimation between immersed and distanced conditions in each of the two emotion conditions separately. Optimistic risk estimation was higher in the distanced fear condition (*M* = 3.46, *SD* = 1.22) compared to the immersed fear condition (*M* = 3.13, *SD* = 1.09), *t*(323) = −2.22, *p* = 0.013 (one-tailed), *d* = −0.25, 90% CI [−0.43, −0.06]. There was no statistically significant difference in risk estimation between the immersed anger and distanced anger conditions, *t*(308) = −1.64, *p* = 0.10 (two-tailed), *d* = −0.19, 95% CI [−0.41, 0.04]. The section below explores the main effect of distancing further by testing whether self-reported fear mediates the relationship between incidental distancing and risk estimation.

#### Exploratory Mediation Analysis

**Figure 3:**
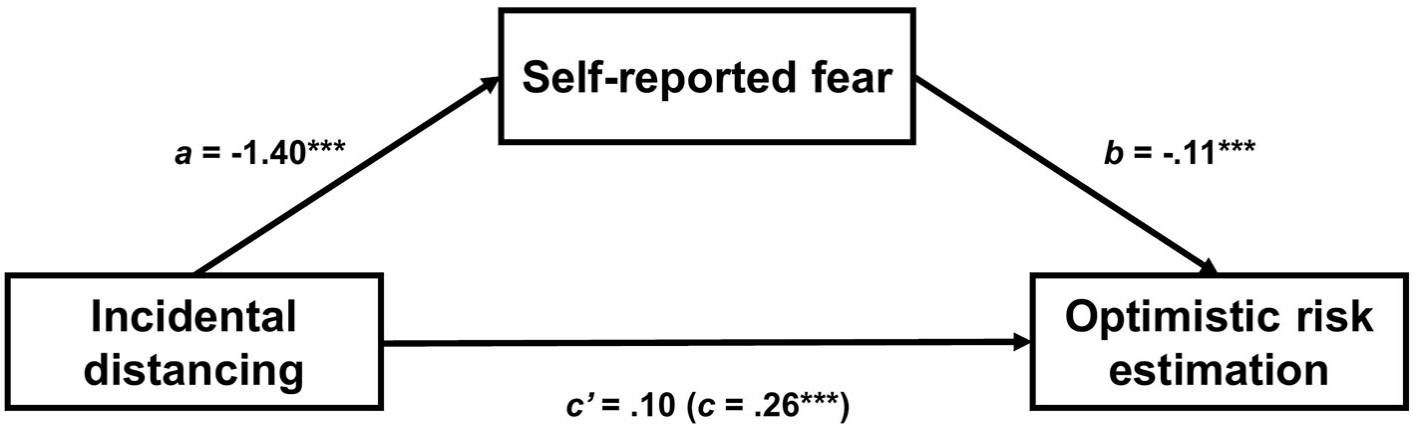
Mediation model in Study 3. Coefficients are unstandardized regression coefficients. The unstandardized regression coefficient representing the total relationship between incidental distancing condition and risk estimation is in parentheses. ***p < 0.001.

Given the main effect of distancing on risk estimation found earlier (section Hypotheses Testing), we performed a mediation analysis to explore whether incidental distancing increased optimistic risk estimation through reduced fear (as measured with the manipulation check). The analysis followed recommendations by Yzerbyt et al. (2018), using the JSmediation package. First, we report the results from the joint significance test of the a-component (a path) and b-component (b path) of the mediation model and conclude mediation if both are significant. Next, we report the boot-strapped estimated size of the indirect effect (*ab*) and its 95% confidence interval. Results indicated that reduced fear, but not anger, mediated the relationship between incidental distancing and optimistic risk estimation. Specifically, both the a and b paths were significant [a point estimate = −1.40, SE = 0.15, *t*(641) = 9.59, *p* < 0.001, b point estimate = −0.11, SE = 0.02, *t*(640) = 4.77, *p* < 0.001], as was the indirect effect (point estimate = 0.16, 95% CI [0.09, 0.23], 5,000 Monte Carlo iterations). The model is illustrated in Figure 3.

### Discussion

In Study 3, we aimed to replicate the findings from the previous two studies by manipulating emotion and distancing. Furthermore, we adjusted our emotion manipulation to the current COVID-pandemic for a more ecologically valid manipulation. We found no support for our hypothesis regarding a moderating role of distancing, nor did we find a main effect of emotion (i.e., fear and anger). However, we found a positive main effect of distancing on risk estimation (but not risk taking). Participants in the distanced condition showed more optimistic risk estimations in a subsequent risk judgment task than participants in the immersed condition. Further exploratory analysis indicated that the effect of distancing on optimistic risk estimation was mediated by reduced fear. In other words, adopting a distant perspective while reflecting on current stressors increased optimistic risk estimation by reducing fear. However, the lack of a control group prevents us from drawing more specific conclusions. We expand on these points in the next section.

## General Discussion

The current study set out to examine how psychological distancing moderates the relationship between fear and risk taking, and anger and risk taking. In Study 1, at low levels of habitual distancing, dispositional fear predicted lower risk taking, whereas dispositional anger predicted greater risk taking. These relationships (fear and risk taking, anger and risk taking) reversed among individuals higher on distancing. Study 2 manipulated distancing and used different measures of dispositional fear and anger. Distancing interacted with dispositional fear but not anger. Replicating the pattern for fear observed in Study 1, the relationship between fear and risk taking was negative for participants who adopted a distanced perspective while reading the risk scenarios, but positive for those who adopted an immersed perspective. Finally, Study 3 manipulated emotions and distancing to examine the impact of incidental distancing from fear and anger on risk preference and risk estimation. While the study found no main effect of emotion or interaction between emotion and distancing on risk preference and risk estimation, exploratory analyses revealed that incidental distancing (across both emotion conditions) increased optimistic risk estimation through a reduction in self-reported fear. This is a relevant finding, as subjective probabilities inform people on what actions they should take, and thus, may shape important life outcomes. Overall, although we find mixed results across the three studies, the results regarding fear reveal a clearer pattern. Distancing moderated the relationship between fear and risk taking the same way in both Study 1 and 2. While we did not observe a moderating effect of distancing in Study 3, distancing increased optimistic risk estimation via reduced fear.

The results contribute to the field by providing important insight into the interplay between psychological distance and emotions in risky judgment and decision making. Previous research has found that distancing is associated with a range of cognitive (Kross and Grossmann, 2012; Grossmann and Kross, 2014; Sun et al., 2018) and affective benefits (Kross et al., 2014; Bruehlman-Senecal and Ayduk, 2015; Nook et al., 2017, 2020; Ahmed et al., 2018; Powers and LaBar, 2019; White et al., 2019). With respect to its emotion-regulatory function, studies suggest that it may be even more effective than its counterpart tactic *reinterpretation* (Denny and Ochsner, 2014). The overall results of the present research provide some evidence that distancing regulates the influence of incidental fear on judgments and decisions involving risk. The influence of incidental fear (Study 1 and 2) and anger (Study 1) on risk taking was reduced and even reversed among the high distancers. More specifically, at high levels of distancing, fear *increased* risk taking. To our knowledge, this is a previously unknown effect. Since we found it in two studies, there is little reason to believe that this is an artifact. Nevertheless, future research is needed to examine how replicable this effect is (i.e., boundary conditions) and what drives it. The measures that we used did not provide much information about the process behind the effect. A previous study has shown that the relationship between fear and risk taking depends on how individuals cognitively frame the situation (Lee and Andrade, 2015). Although Lee and Andrade (2015) did not examine distancing *per se*, the results suggest that the influence of emotions on risk taking depends on how individuals interpret their emotional experiences. Future studies can try to uncover mediators behind the reversal of the relationship between fear and risk taking by using a similar approach to the one we used in Study 3. In Study 3, we observed that a decrease in fear mediated the positive effect of distancing on optimistic risk estimation. As our emotion manipulation check only tapped into fear and anger, future studies should include mediators that tap into other emotions that are typically associated with optimism, such as hope and relief. Studies can also investigate the mental and cognitive processes underlying the unexpected positive relationship between fear and risk. One example is information processing. Appraisal theories suggest that uncertainty-related emotions like fear increase systematic reasoning, whereas certainty-related emotions like anger lead to intuitive reasoning (Lerner and Keltner, 2000; Tiedens and Linton, 2001; Lerner et al., 2015). It would be interesting to examine whether the unexpected positive relationship between fear and risk taking—and the negative relationship between anger and risk taking in Study 1—is explained by a shift from systematic processing to intuitive processing and vice versa. Relatedly, it is possible that distancing regulates the appraisals underlying the predicted effects of fear and anger on risk taking (Lerner and Keltner, 2001). One could therefore test, for example, whether distancing from fear increases risk taking by reducing the level of uncertainty associated with fear.

It should be noted that the effect occurred in decision situations that were characterized by ambiguity. This is relevant since it appears reasonable to expect that reversal effects occur more often in such situations than those that are less ambiguous. Level of ambiguity might therefore constitute a boundary condition for the reversal effect. Indeed, Lerner and Keltner (2001) documented ambiguity with respect to certainty and control as a boundary condition for the predicted effects of fear and anger. Moreover, although the effects in our study were observed in controlled laboratory settings, they could be expected to exist in real-life decision-making situations (e.g., Hodgkinson et al., 1999). Overall, it remains unclear exactly what lies behind these unexpected associations. We hope that our findings will encourage steps toward a more nuanced understanding of how emotion and distancing interact in risky decision making.

### Limitations and Future Research

We would like to highlight several limitations and directions for future research. Overall, we found mixed results with small effect sizes across the three studies. While habitual distancing interacted with both fear and anger (Study 1), manipulated distancing only interacted with fear (Study 2). Study 3 did not find a moderating role of distancing. One possible reason for the mixed results is that we measured and manipulated both emotion and distancing in different ways across the studies. Study 1 looked at habitual distancing from negative events, whereas Study 2 and 3 manipulated distancing. Moreover, overall, we did not find support for our predicted (based on e.g., Lerner and Keltner, 2001; Lerner et al., 2003, 2015; Habib et al., 2015) main effects of fear and anger. This may be attributed to methodological aspects in our studies, as we used slightly different measurements and manipulations. In the one instance where we used the exact measurement used by Lerner and Keltner (2001), we did find a main effect (anger in Study 2). It appears less likely that the null findings can be attributed to power or sample issues. More research is needed to test the replicability of these main effects of fear and anger, and their boundary conditions.

A key strength of this paper is in the multilevel approach used in Study 1 and 2, where participants received the risky decision-making tasks in different domains and frames. However, these tasks do not reflect decision making in real life. Decisions are often made in situations where information about outcomes is unknown. Furthermore, rather than instructing participants to explicitly engage in psychological distancing, decision scenarios can activate psychological distance indirectly by varying the distance of the targets (see Raue et al., 2015). Raue et al. (2015) showed that increasing the psychological distance in risky scenarios eliminated and even reversed the classic framing effects. They interpreted this in terms of a reduction in emotional intensity and a shift from intuitive to deliberate information processing. Our study is the first to test how distance regulates emotional biases in risky decision making. It would be interesting to test whether indirect psychological distance regulates incidental emotions in similar ways.

Moreover, unlike previous studies that have examined the general reappraisal strategy, participants in this study were not explicitly told that the goal was to down-regulate negative emotions through reappraisal. The literature suggests that distancing is an efficient but relatively effortless tactic (Moser et al., 2017) with long-term benefits such as reduced levels of stress (Denny and Ochsner, 2014). There is, however, a need for further research on how distancing impacts risky decision making in emotionally intense real-life situations.

However, studies will also need to examine conditions under which distancing may be ineffective, or even backfire. As noted by Sheppes and Levin (2013), the decision to apply an emotion regulation strategy is a difficult decision in itself. In situations where emotions are known to influence our judgments and decisions in a negative way, it should be advisable to regulate emotions. In other situations, however, it may be less advisable to regulate emotions. Despite potential downsides, we believe that the main function of distancing is not to eliminate emotions, but rather, to help individuals process them.

Finally, there is evidence suggesting that distancing may be less effective in regulating certain emotions. Construal Level Theory (CLT) distinguishes between emotions based on their underlying level of construal (i.e., level of abstractness). For instance, fear constitutes a so-called “low-level” emotion because it is concerned with immediate and visible threats (e.g., seeing a snake while hiking). Anxiety, on the other hand, is a “high-level” emotion because it is concerned with distant and ambiguous threat (e.g., feeling anxious about the possibility of losing one's job in the future). A similar distinction has been made between personal (low-level) and moral anger (high level) (Agerström et al., 2012). Because high-level emotions like anxiety and moral anger necessitate distancing, CLT predicts that distancing may in fact intensify these emotions. Doré et al. (2015) found that use of anxiety-related words following a tragic event increased over temporal and spatial distance. The opposite was found for sadness-related words. Relatedly, Bornstein et al. (2020) found that abstract processing decreased fear and intensified other high-level emotions like guilt. Agerström et al. (2012) found that greater temporal distance increased anticipated intensity of moral anger but decreased the anticipated intensity of personal anger. Although these studies did not use the same manipulations as those used in our study, the pattern of results suggests that distancing might have different effects on different emotions. Thus, future research examining emotion regulation through distancing and decision making should take into account the abstraction level of the emotion, in addition to other appraisals like certainty and control.

## Practical Implications and Concluding Thoughts

The present study points to distancing as a promising tool in organizational settings. For instance, contexts that favor systematic and rule-based decision making might benefit from distancing as a simple tactic to help decision makers avoid excessive risk aversion or risk taking. The idea that a big picture focus can help improve decision making under risk is not new. In fact, in an early paper on the cognitive aspects of risk taking, Kahneman and Lovallo (1993) argued that “a broad view of decision problems is an essential requirement of rational decision making” (p. 20). They further argued that decision makers, particularly managers, tend to adopt a narrow frame of decision problems, failing to place them in broader contexts (Kahneman and Lovallo, 1993). Extending Kahneman and Lovallo's (1993) notion, we believe that one way in which a broad perspective impacts decision making is through the regulation of emotional influences. Distancing can prove effective in situations where fear might lead to excessive levels of risk aversion and where anger might lead to excessive levels of risk taking. Moreover, moving beyond self-regulation, it would be interesting to examine how leaders can regulate employees' emotions and cognitions. Anecdotal reports suggest that employees around the globe may be experiencing high levels of anxiety and pessimism brought by COVID-19 (Jacobs and Warwick-Ching, 2021). It is conceivable that leaders can regulate employees' negative emotions and perceptions by removing them from the “here and now.”

## Data Availability Statement

The datasets presented in this study can be found in online repositories. The names of the repository/repositories and accession number(s) can be found in the article/supplementary material.

## Ethics Statement

The studies involving human participants were reviewed and approved by NSD–Norwegian center for research data. The patients/participants provided their written informed consent to participate in this study.

## Author Contributions

LM and FB contributed to the conception and design of the study. LM collected and analyzed the data and wrote the first draft of the manuscript. Both authors contributed to the article and approved the submitted version.

## Conflict of Interest

The authors declare that the research was conducted in the absence of any commercial or financial relationships that could be construed as a potential conflict of interest.

## Footnotes

^1^https://osf.io/hg358/?view_only=510f9016d0fc47c39488665fda8d14ab

^2^We use the more contemporary label instead of Asian Disease Problem.

^3^The pre-registrations lacked the specification that framing would be used as a control variable. Excluding framing as a control variable from the Study 1 analysis did not significantly change the interaction between distancing and anger but rendered the interaction between distancing and fear insignificant. Excluding framing from the Study 2 analysis did not significantly change any of the two interactions.

^4^Although the Study 1 preregistration included directional hypotheses—which justifies the use of one-tailed tests (Cho and Abe, 2013)—it did not specify whether one-tailed or two-tailed tests would be used. However, Study 2 and Study 3 preregistrations have specified the use of one-sided testing.

^5^Table generated using the tab_model function in the “sjPlot” in R (Lüdecke, 2021).

^6^Plot created using the interact_plot() function in the “interactions” package in R (Long, 2020).
